# Sticky Trap Design Considerations for Entrapping Bed Bugs

**DOI:** 10.3390/insects10060177

**Published:** 2019-06-19

**Authors:** Benjamin A. Hottel, Roberto M. Pereira, Salvador A. Gezan, Philip G. Koehler

**Affiliations:** 1 Center of Biological Control, Florida A&M University, Tallahassee, FL 32307, USA; 2 Department of Entomology, University of Florida, Gainesville, FL 32608, USA; rpereira@ufl.edu (R.M.P.); pgk@ufl.edu (P.G.K.); 3 School of Forest Resources and Conservation, University of Florida, Gainesville, FL 32608, USA; sgezan@ufl.edu

**Keywords:** surface roughness, bed bug trap, sticky trap, aversion, cockroaches

## Abstract

Little evidence has been presented on the usefulness of sticky traps for monitoring bed bugs, *Cimex lectularius*. We examined how the surface roughness around the adhesive of a sticky trap affects both bed bug behavior and adhesive entrapment. In the first assay, bed bugs were placed onto acetate paper discs with different roughness averages (R_a_). Each disc was surrounded by sticky trap adhesive and number of captured bed bugs were recorded. The second assay was set up similarly to the first assay except that the outer portion of the acetate disc had a different R_a_ than the center. In the third assay, bed bugs were placed into circular acetate arenas where they were surrounded by different R_a_ treatments. The number of times the bed bugs contacted the R_a_ treatment but did not cross onto the treatment was recorded. Results of these assays showed that as the acetate surfaces got smoother (lower R_a_), bed bugs were more likely to get trapped in sticky trap adhesives but also less likely to travel across the smoother surfaces they encountered. A sticky trap design with a smooth plastic film around the adhesive was tested in the field to see if it could capture bed bugs in apartments with known bed bug activity. This trap was not only able to capture bed bugs but was also able to detect unknown German cockroach, *Blattela germanica,* infestations. Sticky trap designs with smooth surfaces around an adhesive could be used to monitor not only bed bugs but also German cockroaches.

## 1. Introduction

With the resurgence of bed bugs, *Cimex lectularius* L. (Hemiptera: Cimicidae), in the western world over a decade ago [1,2], a search for effective monitoring and control measures were sought after [3]. A pest control industry survey conducted in 2005 found that 67.9% of pest control companies were using sticky traps as monitors for bed bug infestations [4]; however, former studies have shown that sticky traps were only marginally effective when used as bed bug monitors [5]. In contrast, pitfall or “interceptor” trap designs were found to be more effective than even visual inspections at detecting low level infestations of bed bugs [6,7]. The closest trap design to a traditional sticky trap that has been evaluated has been the Catchmaster BDS (Bedbug Detection System, AP&G Co. Inc., Brooklyn, NY, USA). The Catchmaster BDS has a line of adhesive down the center of the trap. The central adhesive line is surrounded on each side by a row of adhesive dots. The line of adhesive and dots of adhesive can capture bed bugs trying to harbor in the trap. This trap was not found to be as effective as “interceptor” trap designs at detecting bed bug activity [8].

Despite the success observed from using “interceptor” traps for bed bug monitoring, these traps are designed to catch only one pest species within the urban environment. Often, bed bugs are not the only pests encountered in homes, so a monitoring device that can catch multiple pests would be advantageous and cost-effective. Sticky traps, however, are proven effective monitors of cockroaches and spiders [9,10,11,12,13]. Sticky traps have also been observed to catch ants, small flies, mice, beetles, and millipedes [14]. Small modifications to sticky traps have been found to increase the catch rate of some arthropods, such as German cockroaches [15]. Certain design characteristics of cockroach sticky traps may be relevant not only for capturing bed bugs but also other crawling arthropod pests. Because previous studies have shown that bed bugs have difficulty in griping smooth surfaces [16,17], smoother surfaces around the adhesive of a sticky trap may prevent bed bugs from escaping from these traps. No studies have examined how surface roughness characteristics of sticky traps affect bed bug behavior or sticky trap catch rates of bed bugs.

We examined the effects of modifying the surface roughness surrounding the adhesive of a cockroach sticky trap on trapping bed bugs. A commercially available sticky trap with a smooth surface around the adhesive was evaluated for its potential to capture both bed bugs and other arthropods in low-income multiunit housing complexes.

## 2. Materials and Methods

### 2.1. Insects

A bed bug strain collected over 40 years ago by Harold Harlan in Ft. Dix, NJ was used for all laboratory assays. This strain displayed similar behaviors to surface roughness preferences as field collected strains from Florida [18]. Bed bugs were reared using methods described by Hottel et al. [16]. Adult bed bugs were fed one week prior to each assay trial. First instar nymphs were unfed and had hatched within a week prior to being used in each assay trial.

### 2.2. Surface Topography Measurements

A Contour GT-I optical profilometer (Bruker Corporation, Billerica, MA, USA) analyzed the surface topography of various surfaces used in laboratory assays. The surfaces of P600-grit, P2000-grit, and P3000-grit sanded acetate paper (Grafix, Maple Heights, OH, USA) were analyzed. Plain acetate paper, a plain index card (Office Max, Naperville, IL, USA), and the card stock from a Victor^®^ roach glue trap and monitor (Woodstream Corporation, Lititz, PA, USA) were also analyzed. All of the acetate paper surfaces were cleaned with water and paper towels before being tested. The magnification and field of view settings of the optical profilometer were set at 20× and 1×, respectively. This led to a measurement area of 315 × 236 μm. Automated VXI universal imaging with a green light illumination were used for all measurements. Four different samples for each surface were analyzed and the roughness average (R_a_) of each sample was recorded. The R_a_ is the arithmetic mean of the absolute values of the distances between the surface profile and a mean line. The mean line is created so that the area between the surface profile above the mean line is equal to the area bellow [19].

### 2.3. Uniform Rough Surface Sticky Trap Assay

**Figure 1:**
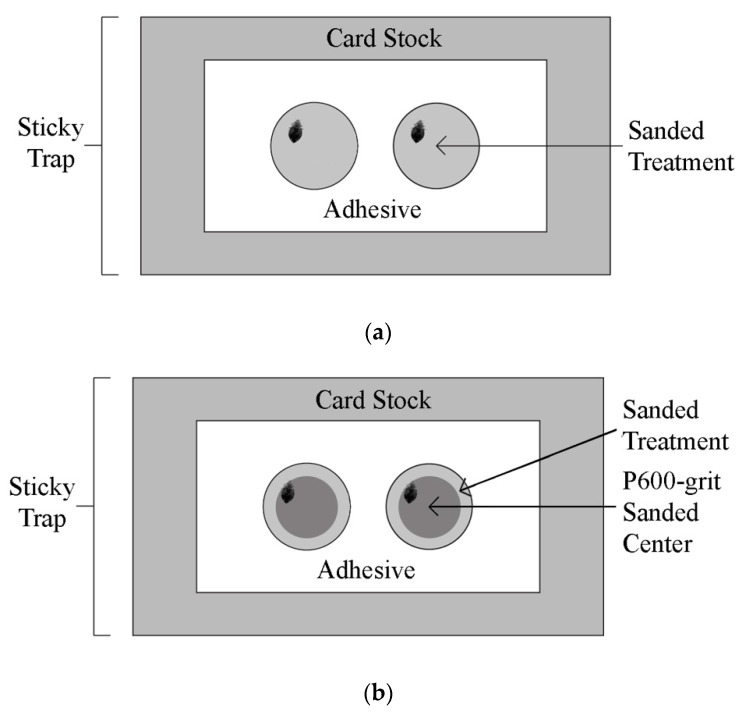
(**a**) Diagram of arena used in uniform sticky trap assay showing a 35 mm diameter index card or a sanded acetate circle (plain acetate, P3000-grit sanded acetate, P2000-grit acetate embedded in a sticky trap, or P600-grit sanded acetate paper). (**b**) Diagram of arena used in dual sticky trap assay with a P600-grit sanded center and a 3 or 7 mm treatment band sanded with P2000-grit sandpaper or P3000-grit sand paper, or not sanded.

An assay was developed to examine the effect of surface R_a_ values surrounding a sticky trap adhesive at capturing bed bugs. Acetate paper or a plain index card was glued (Loctite super glue, Henkel Corporation, Rocky Hill, CT, USA) over the removable paper covering the adhesive of a Victor^®^ roach glue trap and monitor (Figure 1a). Two evenly spaced 37 mm diameter circles were drawn onto the acetate paper or an index card. Acetate circles were sanded with P600-grit, P2000-grit, or P3000-grit sandpaper, or were not sanded. Acetate paper surfaces were cleaned with water and a paper towel after being sanded. Two metal punches 37 mm diameter circles were positioned on both of the circles drawn on top of the acetate paper or index card and a hammer was used to strike the metal punches to form two holes into the cockroach trap and the applied treatment. The discs created from the metal punches, were reinserted into the holes which they once filled. The discs were placed into the holes so that the top portion of the discs (glued acetate paper or index card) was at the same level as the adhesive of the sticky trap. This created a smooth level transition plane from the top surface of the disc to the adhesive surface of the trap. Once the top surface of the disc was level with the adhesive surface, the surrounding removable paper and excess acetate paper or index card were removed from the sticky trap.

Sticky traps with inserted treatment discs (Figure 1a) were placed in a dark windowless room illuminated by one red light bulb. A single male or first instar unfed nymph bed bug was introduced, acclimated, and released into arenas as described above. An observer monitored the bed bugs over the course of the experiment and recorded whether or not the bed bugs were caught within 15 min. Traps that did not catch bed bugs were cleaned with a 70% ethyl alcohol solution and reused up to four times. Individual adult male and first instar nymph bed bugs were tested on each of the five surfaces described above 12 times.

### 2.4. Dual Rough Surface Sticky Trap Assay

The dual rough surface sticky trap assay was similar to the uniform rough surface sticky assay except that instead of having a uniform sanded 37 mm diameter circle, a 7 mm or 3 mm band of varying sanded surfaces was created around a P600-grit sanded center (Figure 1b). The band was sanded with either P2000-grit sandpaper, P3000-grit sandpaper, or not sanded.

Four first instar unfed bed bugs were put in glass vials for each treatment. The glass vials were inverted on top of the center of each circle treatment and left there for 15 min for acclimatization. After 15 min, the vials were removed, and an observer recorded the number of bed bugs caught after an additional 15 min. Arenas were cleaned and reused up to four times if bed bugs were not caught. The experiment was replicated 12 times for each bed bug life stage (male adult vs. first instar nymph), each treatment width (3 mm vs. 7 mm), and on each surface roughness treatment (acetate paper, P2000-grit sanded, and P3000-grit sanded).

### 2.5. Surface Contact Assay

**Figure 2:**
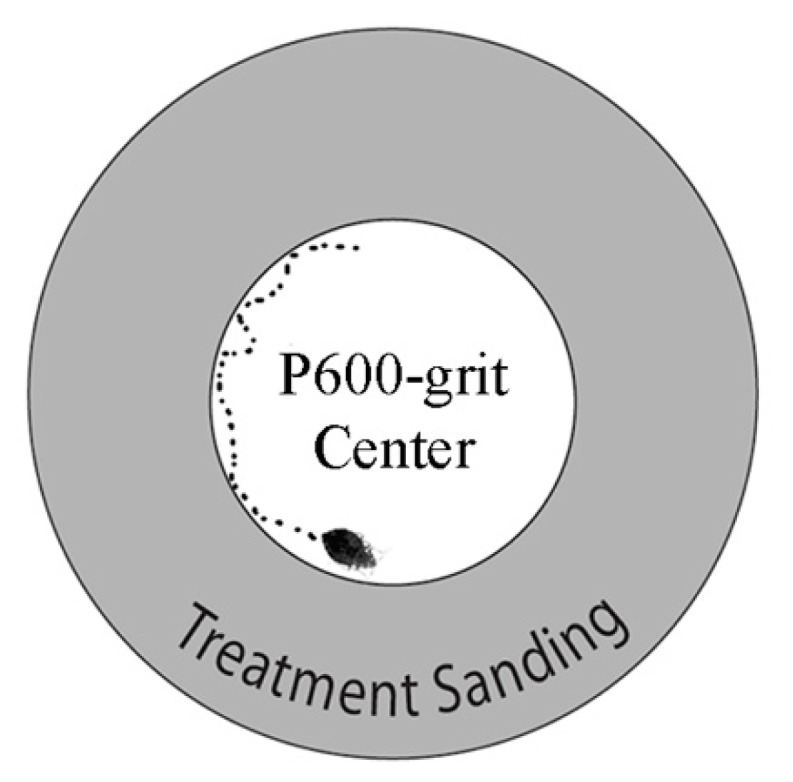
Diagram of arena used in contact assay with an inner 55 mm diameter P600-grit sanded acetate paper at the center of an outer 90 mm circle with different sanded treatments (plain acetate, P3000-grit sanded acetate, P2000-grit sanded acetate, and P600-grit sanded acetate).

In order to examine how bed bugs might interact with modified sticky traps in the field, an assay was also developed to examine the behavior of bed bugs encountering surfaces with different R_a_ values. Test surfaces were prepared by sanding an inner 55 mm diameter circle with P600-grit sandpaper on a 90 mm diameter circle of clear acetate paper. The outer portion of the 90 mm diameter circle that had not been sanded was then sanded with P600-grit, P2000-grit, or P3000-grit sand paper, or left plain. Sanded surfaces were cleaned with water and a paper towel. These treated surfaces were then placed onto a piece of 20.3 × 27.9 cm paper resting in a 550 × 550 mm square Plexiglas arena (Plaskolite, Columbus, OH, UAS) surrounded by clear acetate paper walls forming an arena from which bed bugs could not escape (Figure 2). The arenas were housed in a dark windowless room. Video cameras with embedded infrared cut filters were mounted above each arena tested (Model: FI8910W, Foscam Digital Technologies, Houston, TX, USA). Infrared lights were positioned in the room to provide enough infra-red light for the video cameras to capture detailed bed bug movements. A single bed bug (male, female, or first instar nymph) was placed onto the central 55 mm circles and contained using an inverted 45 mm diameter polypropylene portion container (Dart, Mason, MI, USA). After a 15 min acclimation phase, the cups were removed so that the bed bugs could explore the arena. The video cameras recorded bed bug movement for a maximum of 30 min, but the experiment was stopped if at any point the bed bugs left the 90 mm circle. The time it took each bed bug to leave the 90 mm circle was recorded. In addition, the number of times the bed bugs touched the perimeter of the inner 55 mm circle was counted. These counts will be referred to as “aversion counts.” Aversion counts thus describes the number of times a bed bug decides not to cross a given treatment surface. The experiment was replicated 10 times for each surface treatment (P600 grit, P2000 grit, P3000 grit, or plain) and bed bug life stage (male, female, or first instar nymph) combination.

### 2.6. Smooth Surface Sticky Trap Field Experiment

Field experiments were conducted to verify some of the findings from the laboratory assays. These field experiments were conducted in one and two story low-income multiunit housing complexes located in Tallahassee, FL. These complexes had apartment layouts ranging from one to three bedrooms. Apartments were selected to be included in the field experiment based on positive visual confirmation of bed bugs by a local pest control company servicing the apartments and by one of the authors. A total of seven apartments were selected to be included in the field experiment. No other pest issues were known in these apartments by the landlord or the pest control company. This field experiment was approved by the Florida A&M University Institutional Review Board (IRB) for human subject research (IRB Board Reference # 017-97).

Upon positive confirmation of bed bugs in the apartment during a visual inspection, several M330 Victor^®^ Roach Pheromone Traps (Woodstream Corporation, Lititz, PA, USA) were placed under couches, reclining chairs, and beds. A single trap was placed next to each leg of the furniture. In the case of mattresses that were not placed on a bed frame, two traps were placed next to the mattress where it was in contact with the wall. A total of 79 traps were placed in 6 living rooms and 12 bed rooms over the seven apartments. The mean number of M330 sticky traps that were placed under couches, reclining chairs, or beds in each room of the apartments was 4.4 traps. Traps were inspected for the presence of bed bugs and other arthropods during the scheduled pest control companies’ treatment occurring 7 to 10 days later. These sticky traps were left out on an average of 7.9 days before they were checked. The total number of bed bugs and other arthropods were recorded for each trap.

### 2.7. Statistical Analysis

The binomial capture responses from the uniform sticky trap assay were analyzed using a generalized linear mixed-effects model with a binomial distribution. Life stage (male and first instar nymph) and log_10_-transformed R_a_ values were set as fixed effect categorical and continuous explanatory variables, respectively. Because the traps were reused in some cases described above, a factor that specified each trap was fitted as a random effect predictor variable. An interaction term between life stage and R_a_ was also included if an interaction model was found to have a better fit than the base additive model. Model comparison were evaluated using a chi-square goodness-of-fit test. A Type III Wald test was used to examine the significance of all factors used in the model.

The proportions of first instar nymphs caught in an adhesive surrounded by various surface roughness bands were analyzed using a linear mixed-effects model. Both R_a_ and width were fitted as fixed effects ordinal predictor variables. The specific traps used were set as random effects since they were used multiple times give certain conditions mentioned earlier. An interaction effect between life stage and R_a_ was also included. An analysis of deviance was performed to examine the effects of the fixed effects on deviance and least-squares means comparisons were made between the fixed effect factors.

For the surface contact assay, the number of contacts a bed bug made with the treatment surface was analyzed using a generalized linear model with a Poisson distribution. Life stage (male, female, or first instar nymph) was set as a categorical predictor and roughness average (R_a_) as a continuous explanatory variable. An interaction term between life stage and R_a_ was also included. Over-dispersed data were analyzed using a negative binomial distribution instead of the Poisson distribution given a significant likelihood ratio (LR) test [20]. Analysis of deviance was used to examine the effects of all fitted model terms. Least-squares means comparisons were performed between all explanatory variables.

Proportions of the total number of arthropods trapped in the smooth surface sticky trap field experiment were calculated. A McNemar test for paired data was used on the above counts to determine if positive detection of bed bugs by the M330 trap was successful in detecting bed bugs in a known infestation [21].

R statistical software version 3.1.1 was used to analyze data and create figures (R Development Core Team 2014, Vienna, Austria). The following R packages were used: lsmeans, ggplot2, bear, lme4, car, multcomp, plotrix, pscl, MASS, and gplots.

## 3. Results

### 3.1. Surface Topography Measurements

The mean of the roughness (R_a_) values for each of the six surfaces tested were calculated with reasonable accuracy. The lowest roughness value was observed on plain acetate paper (19 nm ± 0.7) and was followed by P3000-grit sanded acetate paper (114 nm ± 3.9), P2000-grit sanded acetate paper (266 nm ± 13.6), and P600-grit acetate paper (891 nm ± 76.0). The highest roughness values were found on the Victor^®^ Roach Trap cardstock (Woodstream Corporation, Lititz, PA, USA) (25,906 nm ± 980.3) and the index card (25,672 nm ± 1,955.7). A statistically significant difference in roughness was not detected between the Victor^®^ Roach Trap cardstock and the index card (t = −0.116, df = 3, *p* = 0.914), but all other surfaces were statistically different from each other (*p* < 0.01).

### 3.2. Uniform Rough Surface Sticky Trap Assay

**Figure 3:**
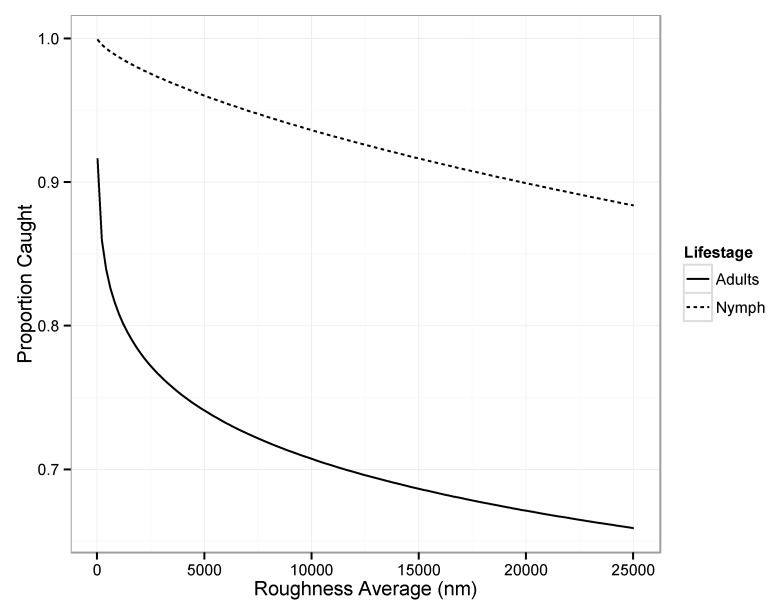
Predicted mean proportion of male and first instar nymph bed bugs, *Cimex lectularius*, caught when placed on a 35 mm diameter sanded acetate paper circle with different roughness (R_a_) values and embedded in a sticky trap arena. The experiment was replicated 12 times for each treatment combination.

The uniform plain acetate (19.3 nm R_a_) surfaces caught the highest proportion of first instar nymphs (0.92) in the sticky adhesive while the index card (25,672.3 nm R_a_) and P600-grit sanded acetate (891.1 nm R_a_) surfaces caught none. The results from the male assay were somewhat similar. The uniform P3000-grit sanded acetate (114.2 nm R_a_) surface caught the highest proportion of male bed bugs (0.83) and the index card (25,672.3 nm R_a_) surface caught none. Examination of the results of the Wald test of the interaction model between life stage (male or first instar nymph) and the log-transformed R_a_ indicated that all factors were significant on explaining capture rates (Table 1). As observed in the fitted model (Figure 3), higher proportions of nymphs were caught, at all values of R_a_, than male bed bugs. The proportion of male bed bugs caught was also noted to be more sensitive to changes in R_a_ than nymphs.

### 3.3. Dual Rough Surface Sticky Trap Assay

Both band width (7 mm or 3 mm) and roughness (19.3, 114.2, 266.1 nm R_a_) had significant effects on the number of first instar nymph bed bugs caught. A linear mixed-effect model without an interaction term was used for the final analysis because a lack of a significant interaction between width and surface roughness was observed in an analysis of deviance (X^2^ = 0.841, df = 2, *p* = 0.657). In the fitted additive model both roughness and width were found to have a significant effect on deviance (Table 2). The highest proportion of first instar nymphs were caught on P3000-grit sanded acetate (R_a_: 114.2 nm; $\bar{x}$ = 0.73; 95% CI: 0.64–0.81) and was statistically significant from both plain acetate (R_a_: 19.3 nm; $\bar{x}$ = 0.50; 95% CI: 0.42–0.60) and P2000-grit sanded acetate (R_a_: 266.1 nm; $\bar{x}$ = 0.46; 95% CI: 0.37–0.55) surfaces (t = −3.793, df = 20, *p* = 0.001; t = 4.499, df = 20, *p* < 0.001). Contrasts between 3 mm and 7 mm widths were also found to be statistically significant (t = −6.014, df = 20, *p* < 0.001).

### 3.4. Surface Contact Assay

**Figure 4:**
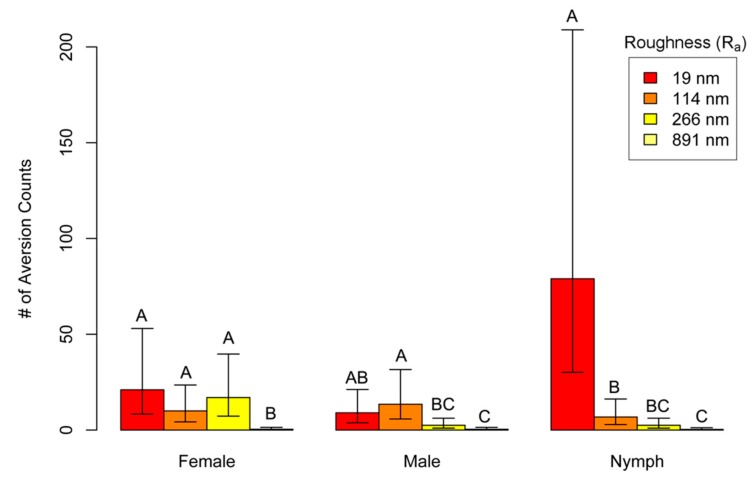
Mean aversion counts for bed bugs that contacted surface treatments when placed on a 55 mm diameter P600-grit sanded acetate circle at the center of a 90 mm circle of different sanded roughness (R_a_) values. Treatments topped by different letters are statistically significant given a least-squares means comparison test at α = 0.05 and 95% CI are shown. The experiment was replicated 12 times for each treatment combination.

Bed bugs displayed various levels of aversion to the surface treatments used (Figure 4). All of the bed bugs eventually left the 90 mm arena except six first instar nymphs on plain acetate (19 nm R_a_) and two on P3000-grit sanded acetate (114 nm R_a_). A likelihood ratio test between the Poisson and negative binomial distribution was statistically significant (X^2^ = 1588.128, *p* < 0.001). Given these results, we concluded that the data was over-dispersed and used the negative binomial model for all further analyses. An analysis of deviance found that roughness (X^2^ = 24.730, df = 3, *p* < 0.001), life stage (X^2^ = 11.238, df = 2, *p* = 0.004), and the interaction between life stage and roughness (X^2^ = 21.986, df = 6, *p* = 0.001) had a significant effect on aversion counts (Table 3). First instar nymphs had the highest number of aversions counts on plain acetate (19 nm R_a_) out of all treatment combination with a mean of 79 (95% CI: 30.2–208.9) counts. The number of aversion counts of first instar nymphs decreased as roughness increased (Figure 4). Female bed bugs displayed a less linear trend than first instar nymphs when examining the interaction of aversion counts and roughness values. Females had similar aversion counts on all surfaces except for P600-grit sanded acetate paper (891 nm R_a_; *p* < 0.001). Male bed bugs also displayed a less linear trend in their aversion behavior than first instar nymphs. Only plain acetate paper (19 nm R_a_; *p* < 0.001) and P3000-grit sanded acetate paper (114 nm R_a_; *p* = 0.001) elicited a significant behavioral response in comparison to P600-grit sanded acetate paper. Mean aversion counts on P2000-grit sanded acetate (266 nm R_a_) and plain acetate (19 nm R_a_) were not statistically significant (*p* = 0.185).

### 3.5. Smooth Surface Sticky Trap Field Experiment

Out of the seven apartments used in the field experiment that were all known to have bed bug activity, bed bugs were caught in sticky traps in 71% (5 out of 7) of these apartments. No statistically significant difference between the 100% of the apartments positively detected for the presence of bed bugs by visual inspections and the 71% of bed bugs found in traps was detected (X^2^ = 3.2, *p* = 0.074). Of the total of 183 arthropods caught in all traps inspected: 83% were German cockroaches, *Blattella germanica* L. (Blattodea: Blattellidae); 14% were bed bugs; 3% were spiders (Araneae); and 1% were varied carpet beetles, *Anthrenus verbasci* L. (Coleoptera: Dermestidae). Of the seven apartments used in the field experiment, 43% (3 out of 7) of them were found to be infested with German cockroaches using the M330 sticky trap.

## 4. Discussion

In the laboratory assays, bed bugs were caught on adhesives depending on the roughness of the surrounding area. Index cards were used to represent the surfaces typically surrounding the adhesives of a commercial cockroach trap. The roughness values of these index cards were found to be statistically similar to the roughness of the card stock used in the standard Victor^®^ roach glue trap and monitor. Male and first instar nymph bed bugs never became entrapped in adhesives when index cards were used. It is unlikely that these standard cockroach sticky traps would be successful in trapping bed bugs in the field.

A comparison of the efficacy of different cockroach sticky traps on catching German cockroaches found that traps that had a smooth plastic film around the adhesive caught more cockroaches than other trap designs [14]. The authors of the study hypothesized that the plastic film reduced the ability of the cockroaches to gain traction on the surface surrounding the adhesive and reduced their chances of escape. In our experiment with bed bugs, we observed a similar finding in that surfaces with lower roughness values than that of an index card were able to catch bed bugs successfully. As the roughness value of the surface decreased, increasingly more bed bugs were caught. As with the cockroaches, this trend is likely attributed to bed bugs’ inability to gain enough grip on the surrounding surfaces to pull themselves out of the adhesive. Bed bugs’ reduced ability to grip smoother verses rougher surfaces has been documented previously [16,17].

While the capability of a trap to physically confine or ensnare an insect is important, behavioral factors can also play major roles in pest-specific trap success [22]. Large differences in the ability of pitfall traps to entrap various stored product beetle species have been previously observed. Much of the variation in catch rate between these species was due to the degree of aversion behavior some of these species had to the top lip of the pitfall trap container. One of the species was so averse to the lip of the pit fall trap that few individuals were caught [22]. A similar aversion behavior was also found with bed bugs encountering smoother surfaces. This aversion behavior was especially pronounced in first instar nymph bed bugs but was less clear with adults.

Maximizing the trap catch of a modified cockroach sticky trap thus involves two conflicting elements: smoother surfaces trap more bed bugs, but smoother surfaces deter more bed bugs from contacting the trap. A comparable situation has been observed with a modified cockroach sticky trap design where an increasing slope at the entrance of a cockroach trap could both deter the cockroaches but also reduce the number of escapes [15]. In our situation, the duel roughness sticky trap assay with two different widths options may shed some light on a compromise between these two opposing elements. The P3000-grit sanded acetate surface (114 nm R_a_) caught the greatest number of first instar nymphs. The reduced catch seen on plain acetate (19 nm R_a_) is likely due to aversion behavior to the smooth surface while the reduced catch seen on the rougher P2000-grit sanded acetate surface (266 nm R_a_) maybe associated with an increased ability of the bed bugs to escape from the adhesive. We are uncertain why there was a greater catch between the 7 mm over the 3 mm band and further investigation is warranted.

Given the results of the uniform rough surface sticky trap assay, the M330 sticky traps were selected to be tested in the field. M330 Victor^®^ Roach traps have a smooth surface plastic film onto which an adhesive is applied. This is in contrast to other sticky traps tested by other authors that have a rough surface base onto which an adhesive is applied [8]. As predicated by the results of the uniform rough surface sticky trap assay done in a controlled laboratory setting, the M330 sticky trap with a smooth surface plastic base was able to detect successfully the presence of bed bugs in field trials. Furthermore, this trap was also able to detect other arthropod pest species. The vast majority of the arthropods caught in these sticky traps were German cockroaches.

The field sites were selected to be in low-income multiunit housing complex in Tallahassee, FL. Low-income multiunit housing complexes have been found to have high infestation rates of both cockroaches and bed bugs. Some of these infestations are unknown to the landlords and residents. In Boston, MA a survey of low-income multiunit housing residents found that 47% and 8% of apartments had cockroach and bed bug activity, respectively [23]. These estimates are probably low since at least one study reported as many as 53% of low-income residents unknowingly having bed bug infestations [24]. In New Jersey, 26% of low-income multiunit housing apartments were found to be infested with bed bugs using pitfall traps and visual inspections. Of these infested apartments, 71% of the infestations were unknown to landlords [25]. In our study, none of the detected German cockroach infestations were known to the landlord or the pest control company servicing the units.

Both German cockroaches and bed bugs have been shown to be able to spread to neighboring apartment units [25,26,27]. Therefore, it is important to find these unknown infestations and take appropriate management strategies so that they do not spread any further. Pest control companies could use M330 sticky traps during bed bug inspections not only to detect potential bed bug infestations but also German cockroach infestations. The same idea could be used during German cockroach inspections in a residence and allow the pest control company to also detect bed bug infestations.

More field research is warranted to examine a sticky trap design with a modified R_a_ value to reduce potential aversion behavior by wandering bed bugs. This modification could lead to higher trap catches and an overall more effective monitoring device. Other factors could increase the trap catch efficiency that were not considered in this study. For example, the traps in this study were only placed out for an average of 7.9 days and the number of traps placed per room was 4.4 traps, on average. Increasing this duration and the number of traps placed per room would likely lead to a higher positive detection rate as has been shown in pitfall monitoring studies with bed bugs [8,24,25]. The number of apartments tested should also be increased in future studies. The number of apartments monitored for the current study was limited by the cases of confirmed bed bug infestations throughout all of public housing facilities in Tallahassee, FL. A larger study would need to be conducted in an environment with higher numbers of infested residences. Improved monitoring for key pests in low-income communities, such as bed bugs and German cockroaches, may lead to more successful management programs, improving community health and wellbeing.

## 5. Conclusions

Current bed bug monitoring traps use pitfall trap designs to entrap bed bugs. These traps are not designed to catch other crawling insect pests such as German cockroaches. Making the surface roughness around the adhesive of a sticky trap smooth can allow the trap to capture both bed bugs and German cockroaches. This will allow pest management professionals to monitor for both bed bugs and German cockroaches at the same time.

## Figures and Tables

**Table 1:** Factor influencing uniform rough stick trap model.

Factor	X^2^	d.f.	*p*-Value
Life stage	4.805	1	0.028
log(R_a_)	11.035	1	*p* < 0.001
Life stage: log(R_a_)	4.795	1	0.029

**Table 2:** Analysis of Deviance table for the duel rough sticky trap model.

Factor	X^2^	d.f.	*p*-Value
R_a_	23.418	2	*p* < 0.001
Width	36.172	1	*p* < 0.001

**Table 3:** Analysis of Deviance table for the aversion model.

Factor	X^2^	d.f.	*p*-Value
Life stage	11.238	2	0.004
R_a_	24.730	3	*p* < 0.001
Life stage: R_a_	21.986	6	0.001
